# Rudimentary horn pregnancy, a differential diagnosis of an intraabdominal pregnancy: a case report

**DOI:** 10.1186/s13256-023-03882-5

**Published:** 2023-05-12

**Authors:** Semtama Bidiga, Kiwango Henry, Onesmo Augustino, Fridolin Mujuni, Dismas Matovelo, Edgar Ndaboine, Albert Kihunrwa, Richard Kiritta

**Affiliations:** Department of Obstetrics and Gynecology, Well Bugando School of Medicine, Catholic University of Health and Allied Sciences, Mwanza, Tanzania

**Keywords:** Pregnancy, Rudimentary horn pregnancy, Abdominal pregnancy, Laparotomy

## Abstract

**Background:**

Rudimentary horn pregnancy is a rare life-threatening obstetric condition with clinical and sonographic presentation resembling that of an abdominal pregnancy. Preoperative diagnosis of advanced rudimentary horn pregnancy is difficult and cases are often identified incidentally during laparotomy for a presumed abdominal pregnancy.

**Case presentation:**

We report a case of a 29-year-old African woman, gravida 2 para 1 at 28 weeks of gestation complaining of epigastric pain for 7 days with no other associated gastrointestinal or genitourinary symptoms. On examination, she had normal vital signs and an enlarged abdomen sized at 33 cm with unremarkable fetal lie and presentation. She had normal laboratory blood results with an ultrasound revealing an abdominal pregnancy of 28 weeks. The informed decision for conservative management was planned after informing of the benefit and risks of early termination versus conservative management, however, with worsening symptoms an emergency laparotomy had to be performed in which a left unruptured rudimentary horn pregnancy with a viable fetus was identified incidentally and delivery of the fetus followed by surgical excision of the horn was done. The postoperative period was uneventful, and the patient was discharged home with her newborn.

**Conclusion:**

Rudimentary horn pregnancy is very rare and often indistinguishable from an abdominal pregnancy in advanced gestation age. First trimester ultrasound is by far the only noninvasive sensitive diagnostic modality for rudimentary horn pregnancy. Laparotomy with horn excision remains the standard of care for advanced rudimentary horn pregnancy.

## Introduction

Rudimentary uterine horn is a congenital anomaly resulting from incomplete unilateral Müllerian duct development and incomplete fusion with a normal contralateral side. Rudimentary horn pregnancy (RHP) is an extremely rare event that is difficult to diagnose due to a lack of specific clinical presentation and a decreased ultrasonography sensitivity as pregnancy advances. Under a good sonographer, first trimester ultrasound is an ideal tool to make the diagnosis of RHP; however, as is often the case for many women in developing countries such as Tanzania, ultrasound is done late, often upon onset of certain complications. Though magnetic resonance imaging (MRI) is more sensitive in diagnosing extrauterine pregnancy in all gestations, its use is often not only limited by conflicting study results on associated adverse pregnancy effects and availability, especially in low- and middle-income countries, but also by a lack of awareness and clinical suspicion of the existence of RHP even in the developed world. We present a discussion of what we believe is the first reported case of rudimentary horn pregnancy from East Africa focusing on clinical presentation, diagnostic challenges, and management of a viable advanced rudimentary horn pregnancy in resource-limited settings.

## Case report

A 29-year-old African woman with her second pregnancy at 28-week gestation was admitted at Bugando Medical Centre (BMC) obstetrics ward as a referral case from a lower-level health facility with a provisional diagnosis of abdominal pregnancy. She presented with mild sharp epigastric pain for 7 days with no other gastrointestinal or genitourinary-related symptoms. Her vitals during admission appeared normal, with a recorded blood pressure of 126/83 mmHg, pulse rate of 91 beats per minute, respiratory rate of 18 breaths per minute, and an axillary temperature of 37 °C. Abdominal assessment showed a distended abdomen sized at 33 cm, unremarkable fetal lie and presentation, and a fetal heart rate of 132 beats per minute by a bedside Doppler fetoscope.

An urgent ultrasound revealed an intraabdominal pregnancy with a live fetus at 28 weeks of gestation. Her hemoglobin was 10.5 g/dl, blood group A Rh^+^, and normal clotting profile. After counseling on the benefit and risks of early termination versus conservative management, a decision for conservative management was reached and the patient was prescribed corticosteroid for fetal lung maturation and intravenous magnesium sulfate for neuroprotection.

**Fig. 1:**
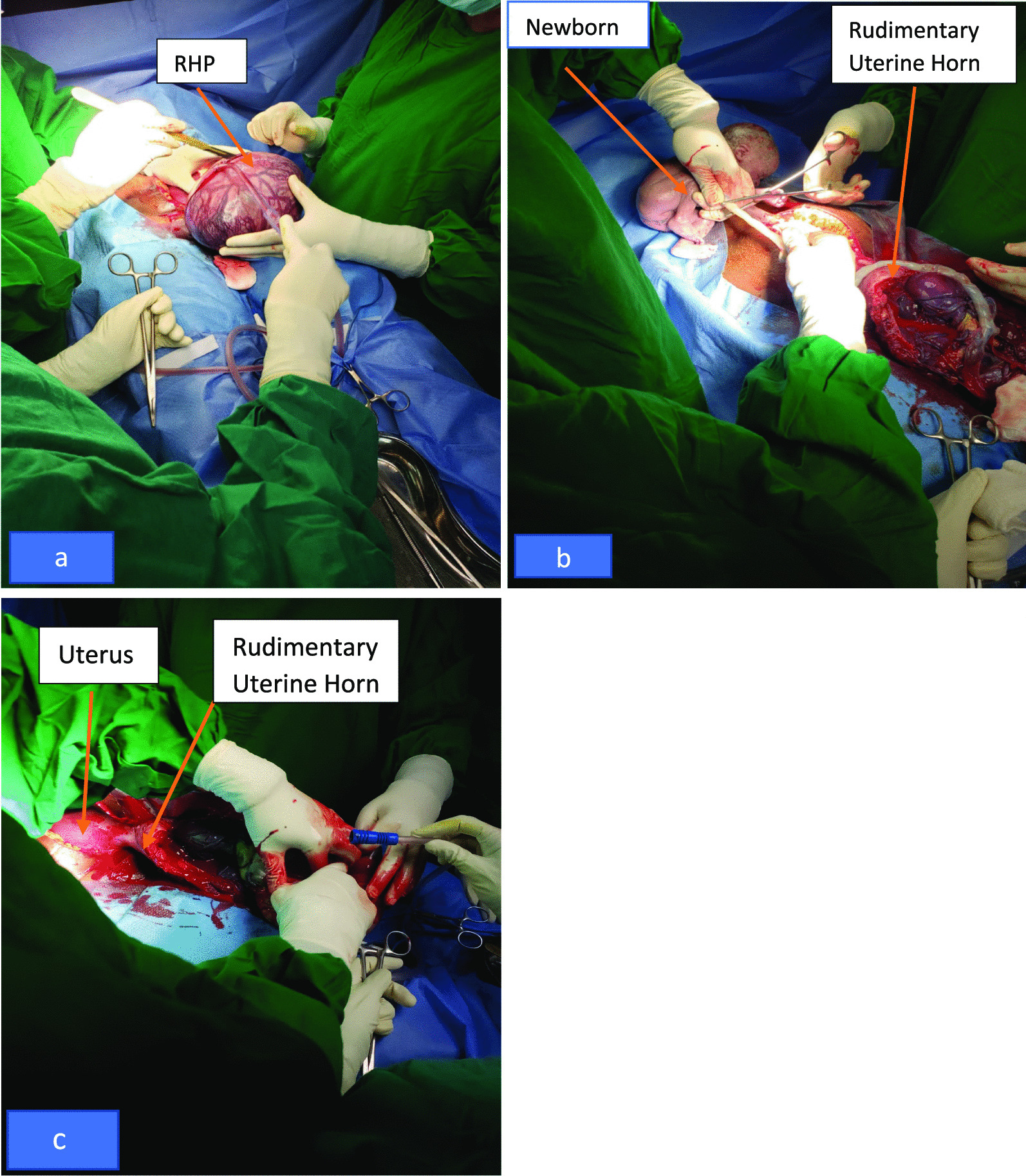
**a** A highly vascularized rudimentary horn, **b** A 1050 g baby delivered from a rudimentary uterine horn, **c** Main uterine body with an incised placenta containing rudimentary horn

On the third day in the ward, epigastric pain worsened with slight tenderness on the epigastrium, no rebound tenderness, and an audible fetal heart rate of 142 beats per minute by a Doppler fetal scope could be heard. From these findings, an emergency laparotomy was done and intraoperatively, a left communicating rudimentary horn pregnancy was found, from which a 1050 g female baby who scored 5 and 7 at first and fifth minute, respectively, was delivered (Fig. 1a–c). The placenta was then removed and surgical excision of the rudimentary horn was done, hemostasis was achieved, and the abdomen closed in layers. Her postoperative period was uneventful and the patient stayed for 40 days in kangaroo mother care (KMC) and was discharged with a baby weighing 1500 g.

## Discussion

Rudimentary horn is a rare congenital uterine anomaly resulting from incomplete unilateral Müllerian duct development and incomplete fusion with a normal contralateral side. Rudimentary horn pregnancy is an extremely rare cause of extrauterine pregnancy with a reported prevalence ranging from 1:76,000–150,000 of all pregnancies [1, 2]. Its clinical presentations echo that of abdominal pregnancy, whereby patients are often asymptomatic in early gestation with the onset of abdominal pain in later gestation that worsens as pregnancy advances [3], as was observed in our presented case.

Both rudimentary horn and abdominal pregnancies infrequently progress to full-term gestation [4, 5]. Rudimentary horn pregnancy (RHP) is often diagnosed intraoperatively while already ruptured due to a lack of distinct clinical and radiological findings [3, 6]. Though a majority of studies suggest using ultrasound as the initial imaging modality to diagnose RHP by noting the sonographic findings of the rudimentary horn, such as a heart-shaped uterus as often seen in asymmetrical bicornuate uterus, absent visual continuity between the cervical canal and lumen of the pregnant horn, and the presence of myometrial tissue surrounding the gestational sac in the first trimester [7], it should be noted that these findings are difficult to elicit as pregnancy advances and the rate of misdiagnosis becomes higher, with reported ultrasound’s sensitivity of 26–33% in advanced gestation [6]. As is often the case in most women in developing countries such as ours [8, 9], both delays in starting prenatal care and lack of routine first trimester obstetrics scanning often result in a missed opportunity for early diagnosis of some of the pregnancy abnormalities, as is seen in this case in which the first ultrasound was done after the onset of abdominal pain at 28 weeks and in which RHP could not be differentiated from an intraabdominal pregnancy sonographically.

Magnetic resonance imaging (MRI), laparoscopy, and/ or hysteroscopy are imaging techniques with high positive predictive value in diagnosing extrauterine pregnancy including rudimentary horn and abdominal pregnancies [10]. MRI can be used in all trimesters, however, the use of gadolinium MRI during pregnancy is not recommended due to reported risks including embryopathy, congenital malformations, early pregnancy loss, intrauterine growth restriction, stillbirths, and neonatal death [11]. Moreover, the use of MRI is often limited by its low availability and high cost in low-resource countries [12]. Lack of suspicion of the possibility of having RHP in the index case coupled with ultrasonographic findings suggestive of abdominal pregnancy hindered the use of MRI to ascertain the final diagnosis despite its availability in our facility.

Similar to other types of ectopic pregnancy, depending upon the gestational age, RHP can either be managed medically or surgically (laparotomy and/or laparoscopic) [3, 6]. Laparoscopic surgery, when performed on asymptomatic women in early gestation, may lead to effective surgical results [10, 13, 14]; however, laparotomy continues to be the cornerstone of treatment for both ruptured and unruptured rudimentary horn pregnancies in all trimesters [3, 15], especially with advanced pregnancies as was observed in our case.

## Conclusion

The rarity of rudimentary horn pregnancy coupled with the lack of distinct clinical presentation and reduced sonographic sensitivity as pregnancy advances make it difficult for clinicians to differentiate advanced rudimentary horn pregnancy from an abdominal pregnancy. First trimester ultrasound should be performed on all pregnant women to ascertain pregnancy location. MRI is an investigation of choice to distinguish RHP from abdominal pregnancy in advanced gestation. Laparotomy aimed at evacuating the horn followed by its resection is the standard mode of treatment for advanced RHP.

## Timeline

The patient was admitted on 20 March 2022, and investigations and management were initiated upon admission. She underwent an emergency laparotomy on the third day post-admission, and postoperative care continued for 40 days after surgery, as she was nursing her baby in the premature unit. Preparation of this case report took 3 months, during which the case was presented at an obstetric conference at Bugando Medical Centre and to the ethical committee after obtaining the patient’s consent.

## Data Availability

Not applicable in this case report.
